# Fulton County Medical Society

**Published:** 1916-02

**Authors:** 


### FULTON COUNTY MEDICAL SOCIETY

(Regular Meeting February 17, 1916.)

Dr. C. E. Dowman read a paper on Treatment of Fractures of the Base of the Skull.

Dr. W. P. Nicolson said that in his experience operations for fracture of the base of the skull had not been satisfactory, but that it should be done. No one can know just what has taken place either in the bone itself or within the skull cavity unless he sees. When the fracture occurs in other parts, decompression operations should be done when a man becomes unconscious after injury without waiting for high systolic blood pressure. Middle meningeal hemorrhage means pressure upon the brain and must be relieved surgically. Epidural hemorrhage and clots are easy to handle and the results are brilliant. A patient has more chances with an operation than without, because when the work is carefully done, there can be no harm, and when it is not done at all, grave injuries may be left unattended· He related several cases in support of his remarks.

Dr. J. L. Campbell agreed that in cases of fractures of the skull where there is hemorrhage, operation should be done quickly, but in cases where there were immediate unconsciousness and flow of cerebro spinal fluid from the ears indicating a true fracture of the base, there is doubt as to the value of operation. The shock is already very severe and the work may simply add to it.

Dr. D. F. Winn said that this subject was growing in importance because of the many injuries that were coming to the hospitals because of automobile accidents. He believed in prompt oprative relief for all fractures with hemorrhage and pressure symptoms. He urged that case records be kept with greater care so as to throw guiding light upon all the symptoms. Blood pressure, time of unconsciousness, vomiting, pupilary reaction, paralyses and skin reflexes should all be noted and observed frequently in the course of the case.

Dr. E. L. Griffin said that a man in continuing unconsciousness after an injury is likely to be in a lethal state all the time and the operation would not harm him further. Whether it would help or not is a question for experience to answer. But in a case resuming consciousness and then becoming unconscious once more, there is surely hemorrhage and pressure and he should have quick surgical relief, no matter what the other symptoms were. There is no use waiting for anything else. The localization of the field of operation is difficult especially in unconscious cases. When there is no definite guide, he uses the right side.

Dr. O. O. Fanning related a case in which operation had been delayed for two years after an injury to the head. The man had "spells" like fits and came here for relief. Operation removed a mass of infiltrated material from the lower occipital region and the man recovered perfectly.

Dr. J. F. Denton said that he did a decompression operation yesterday and while it was too soon to announce results, there had been great intracranial presure and the patient was manifestly improved by releasing it. He has noted that among the insane there are many cases of the injured who have recovered without operation.

Dr. Dowman closed and said that cerebral concussion is a doubtful condition. There are always anatomic changes somewhere in the brain mass and they often take the form of minute petechial hemorrhages. Of course severe cases are likely to die anyhow and when they have gone long enough for the systolic pressure to fall showing that compensation has gone, the patients will die. Subtemporal decompression is the operation of choice, the bone is thin and much of it may be removed without harm to the patient. Large areas can thus be reached. The dura should be opened and left open for drainage. The middle menigeal artery is usually injured in fractures of the base as well as of the parietes.

Dr. D. F. Winn read a paper, A Review of 132 Consecutive Operations for Fibroid Tumors of the Uterus.

Dr. J. F. Denton said that he had not followed up his fibroid cases as carefully or extensively as had Dr. Winn. At Grady hospital where there were almost innumerable fibroids among the colored people, there were so many other interesting complications that the statistics had escaped his attention. A Cyst of the Round Ligament, A Large Lipoma of the Abdominal Wall and An Ectopic Pregnancy of long duration were recent events that would show what he meant. Another case presented signs of pregnancy and a mass appearing like a fetal head presented itself in the vagina. When an interne attempted to deliver it, a condition resembling an everted uterus resulted. The uterus could not be differentiated from the mass until it was isolated by adbominal section and a fibroid removed. Recovery was complete. The statistics are of greatest value and should be studied as Winn has put them forth.

Dr. J. L. Campbell asked as to the heart conditions in these cases.

Dr. Nicolson asked as to the proportion of colored cases.

Dr. Winn closed and said that his mortality had been 9. The heart conditions had not been specially noted. The racial proportion was white 36, black 96.

Dr. J. C. Johnson read a paper, Lest We Forget. It appears elsewhere in the Journal-Record.

Dr. E. C. Davis said that the subject was fascinating and that the essay called to mind a great wealth of things for us to consider and ponder over. We have accepted many traditions in surgery without criticism and analysis. Therapeusis is sent to us by wholesale drug houses and cleverly delivered by detail men in ways sometimes convincing against better judgment. He agrees with the essayist that the internists should not be set aside by the surgeons in enteric matters and says that consultation should be more frequent.

Dr. R. T. Dorsey said that now-a-days we are not following the traditions as we once were but that doctors are studying and judging. We do not accept a man's dictum simply because he is or has been a great man. Osler and Cabot both say that we are wrong in our diagnosis in from 50 to 60% of our cases, but he doesn't believe it. If we try to announce all the contributory causes of death, the laboratory findings will show we are lacking in many things, but as to the precipitating cause, which would indicate that we knew what we were trying to combat, we are right in a large majority of our cases. In expert testimony the examiners do not want us to tell the truth and will prevent our saying all of it by their technicalities. We should insist upon telling it all or upon not going on the stand. Surgeons are more exact in their diagnosis and treatment than the internists and in primary diseases of the stomach, they are more successful.

Dr. E. C. Cartledge said that there were some surgical cases of the enteric tract that got away from the internists and recalled the case of a prominent Atlanta physician, himself an internist, who thought he had gastric disease but was cured by appendectomy after many years of distress. After all, the general practitioner is the one who can weigh both sides and is able to judge them accurately more often than the man whose attention is too closely held by one line of events.

Dr. G. M. Niles said that whatever Dr. Johnson said was worthy of great attention because he spoke with authority. The reading by the partially informed public of articles from the government on public health matters is attended with risk because of wrong conceptions likely to be formed. More explanations are needed to inform a layman than a careful article for the profession is likely to have. Ask a layman what his impressions are after reading the best of these and note how far away he has lead himself. As to surgery and internal medicine, they both have their functions and it is to be remembered that peace has her victories as well as war.

Dr. G. C. Mizell agreed with the writer as to the sending indiscriminately the public health reports to the newspapers.

Dr. Johnson closed and said that we must soon take a definite stand and tell all the truth in the practise of medicine. There must be justice between the public and the profession.

Dr. S. T. Harris exhibited two men and remarked upon the earning power as a result of artificial pneumothorax. Each man had cavities in the lungs and was rundown and unable to work. A short time after the treatment he was feeling well and for many months now, he has been earning his living. One man gets $125 a month and the other $5 a day. Plates were shown portraying the cavities.

Dr. E. P. Merritt presented a case of secondary syphilis in a young man. The rash has been pustular but was rapidly improving after one dose of arseno benzol .4 gm.

Dr. H. I. Battey showed photographs of an ovarian cyst which he said weighed 200 pounds. He had not been able to find a record of so large a one.

